# Antecedents of perceived teacher work ability: a comprehensive model across work and non-work domains

**DOI:** 10.3389/fpsyg.2025.1557456

**Published:** 2025-02-21

**Authors:** Petr Hlado, Libor Juhaňák, Klara Harvankova

**Affiliations:** Faculty of Arts, Masaryk University, Brno, Czechia

**Keywords:** work ability, perceived teacher work ability, job demands, job resources, teacher burnout, work-life conflict

## Abstract

**Introduction:**

This study investigates the antecedents of perceived teacher work ability, a critical construct for addressing challenges associated with extending working lives and maintaining sustained professional engagement in the teaching profession. Grounded in the job demands-resources (JD-R) model, this cross-sectional study investigates the relationships between job demands (quantitative, cognitive, and emotional), job resources (supervisor support, coworker support, and autonomy), burnout, and work-life conflict in shaping perceived teacher work ability.

**Methods:**

Data were obtained from 841 Czech primary and lower secondary school teachers (86.1% female) with a mean age of 45.9 years (SD = 10.8). The full SEM model was developed and estimated based on the proposed hypotheses.

**Results:**

The findings suggest that burnout is the most significant antecedent of perceived teacher work ability, with quantitative and emotional job demands indirectly influencing it through burnout. While supervisor and coworker support directly enhance perceived teacher work ability, autonomy primarily alleviates quantitative and emotional job demands, thereby indirectly mitigating burnout. Work-life conflict partially mediates the relationship between job demands and burnout but does not directly impact perceived teacher work ability.

**Discussion:**

These results contribute to a deeper understanding of the interplay between job demands, job resources, and burnout in enhancing teachers' capacity to meet the multifaceted demands of their profession effectively.

## Introduction

In recent decades, the demographic composition of the teaching population has undergone significant changes. The aging of the teaching workforce is a widespread and critical phenomenon observed across North and Latin America, as well as in many European countries (European Commission, 2023a; UNESCO, 2024). The average age of Czech teachers in primary and lower secondary schools is 46 years, with over 65% aged 50 or older (Czech School Inspectorate, 2023). Due to an aging workforce, educational systems are either already facing or will soon encounter significant teacher shortages, which pose a range of challenges for both educational policies and systems (UNESCO, 2024). At the individual level, research indicates that such shortages can lead to a considerable increase in the workload of current teachers (European Commission, 2023c). In response to these demographic shifts, there is a need to develop and implement target support strategies that address the distinct needs of teachers across all age groups, aimed at enhancing their capacity to perform job tasks effectively and improving retention within the profession.

In efforts to extend working lives, growing attention has been directed toward the construct of work ability (WA), defined as an individual's ability or perception of their ability to meet job demands and complete required tasks (Ilmarinen et al., 1997). This construct was proposed in the 1980s by researchers at the Finnish Institute of Occupational Health to assess whether individuals can continue to meet the physical and psychosocial requirements of their profession (Ilmarinen et al., 1991). Ilmarinen et al. (2005) emphasizes that high WA is crucial for promoting longer work life and preventing early retirement. Conversely, low WA results in a range of unfavorable employment outcomes. For instance, it is linked to a decline in work performance and productivity (Van Den Berg et al., 2009), short-term and long-term absenteeism (Alavinia et al., 2009; Sell, 2009), low job satisfaction (Milosevic et al., 2011), the decision to leave the profession prematurely and early retirement (McCarthy et al., 2024; McGonagle et al., 2015; Vertanen-Greis et al., 2022).

Since its establishment, WA has primarily been assessed based on evaluations of an individual's health and functional capacity. However, recent critiques of this construct have led to a differentiation between objective and perceived WA (Brady et al., 2020; Cadiz et al., 2019; Freyer et al., 2019; McGonagle et al., 2015). Objective WA is based on assessing the employee's health and functional limitations (McGonagle et al., 2022). In contrast, perceived WA refers to the worker's self-perception or subjective assessment of their ability to continue working in their current job, considering the job characteristics and available resources (McGonagle et al., 2022). Despite substantial research focusing on objective WA (Cadiz et al., 2019), perceived WA has received considerably less attention (Guidetti et al., 2018; Hlado and Harvankova, 2024; Magnavita et al., 2024; McCarthy et al., 2024; McGonagle et al., 2015, 2022; Selander et al., 2023). Furthermore, research on perceived WA has predominantly focused on non-teaching professional populations, highlighting the need for more targeted studies on teachers' WA.

This study focuses on teachers' perceived work ability (PTWA), conceptualized as the subjective evaluation of their physical and mental capacity to meet job requirements and effectively navigate the diverse physical, cognitive, interpersonal, emotional, and organizational demands inherent in contemporary teaching roles (Hlado and Harvankova, 2024; McCarthy et al., 2024). The goal of this study is to provide a more nuanced understanding of the antecedents of PTWA while advancing theoretical frameworks, informing future research, and offering recommendations for planning and implementing PTWA interventions. We surveyed a large and diverse group of Czech primary and lower secondary school teachers to achieve this goal. The Czech Republic is particularly suited for this research because it is one of the OECD countries with a high proportion of aging teachers. Nearly one-third of teachers in the Czech Republic (30.5%) are aged 55 or older, considerably exceeding the EU average of 24.5% (European Commission, 2023b).

We contribute to the literature by comprehensively examining the antecedents of PTWA. The job demands-resources (JD-R) model and WA research (Bakker et al., 2005; Cadiz et al., 2019; Hlado and Harvankova, 2024) have identified three key categories that play a significant role in the depletion of perceived WA: job demands, job resources, and burnout. Therefore, we investigate how job demands (quantitative, qualitative, and emotional), job resources (supervisor, coworker support, and autonomy), and teacher burnout interact to affect PTWA. In addition to replicating established predictors of perceived WA, we contribute to the literature by exploring an additional determinant—work-life conflict—which is not included in the JD-R model (Demerouti et al., 2001; Demerouti and Bakker, 2011) and remains underexplored. Thus, the present study empirically tests the underlying psychological processes captured in the JD-R model, which has been expanded to include a non-work domain. The contribution of our study lies in its holistic approach, investigating the effects of the model variables on PTWA and the complex interrelationships among them.

## Theoretical background and hypotheses development

The construct of WA initially emerged without a theoretical grounding (Cadiz et al., 2019). A proper theoretical framework for investigating WA, particularly PTWA, is provided by the JD-R model (Cadiz et al., 2019; Demerouti et al., 2001; Demerouti and Bakker, 2011; Schaufeli, 2017; Schaufeli and Taris, 2014). Although the JD-R model was not designed initially to address WA, it has been effectively applied in this context. Perceived WA can be integrated into the JD-R model as an outcome that is positively influenced by job resources and negatively affected by job demands (Brady et al., 2020; Cadiz et al., 2019; Hlado and Harvankova, 2024; McGonagle et al., 2014, 2022; Schaufeli and Taris, 2014).

The JD-R model (Bakker and Demerouti, 2007) asserts that every job encompasses various types and levels of job demands that can impede employee functioning while also highlighting the role of job resources that can support and enhance employee performance and wellbeing. Job demands refer to the physical, psychological, social, or organizational characteristics of a job that require sustained physical or mental effort and are associated with physical and psychological costs (Demerouti et al., 2001). Examples of job demands in the teaching profession include work overload, administrative tasks, time pressure, multitasking, responsibility, disruptive student behavior and misbehavior, difficulties in communication and cooperating with parents (Hlado et al., 2020; Hlado and Harvankova, 2024; Schaufeli and Taris, 2014). Conversely, job resources are the physical, psychological, social, or organizational factors that can be functional in achieving work goals, reducing job demands and associated physiological and psychological costs, and encouraging personal growth and development (Schaufeli, 2017). In the context of teaching, job resources include job control, a supportive social climate, constructive feedback, supervisory coaching, student appreciation, and professional development opportunities (Hlado and Harvankova, 2024; Schaufeli and Taris, 2014).

The JD-R model delineates two independent psychological processes: health impairment and motivation (Bakker and Demerouti, 2007; Demerouti and Bakker, 2011; Schaufeli and Taris, 2014; Taris et al., 2017). First, the health impairment or energetic process posits that when job demands exceed individual capabilities and are accompanied by low levels of job resources, it can result in the depletion of mental and physical energy. This depletion, in turn, may contribute to developing health-related issues and adverse job-related outcomes, including reduced WA. The JD-R model in the health impairment process suggests that the relationship between job demands and outcomes mediates burnout through the gradual depletion of mental resources. Second, the motivational process posits that job resources have motivational potential, fostering high work engagement and positive job-related outcomes (e.g., work performance, innovativeness, organizational commitment, service quality, and intention to stay).

Alongside the main effects of job demands and resources, the JD-R model specifies how job demands and job resources interact and predict important outcomes (Bakker et al., 2005, 2007; Bakker and Demerouti, 2007; Demerouti and Bakker, 2011). More specifically, the buffering hypothesis postulates that high levels of job resources can mitigate the adverse effects of job demands on job strain, including burnout and other outcomes.

### The effect of job demands

Job demands were linked to physical and psychological costs incurred by teachers in meeting job tasks and challenges (Hlado and Harvankova, 2024). Job demands are recognized as crucial determinants affecting WA (Brady et al., 2020; Kunz and Millhoff, 2023). Prior research has shown that various physical, mental, psychosocial, and emotional job demands negatively impact WA across general adult populations as well as perceived WA in the teaching profession (Cadiz et al., 2019; Hlado and Harvankova, 2024; Truxillo et al., 2020). Among teachers, the job demands affecting WA include aspects like a poor indoor environment, workplace noise, high workload (which involves administrative tasks and supervision of students during breaks), homeroom teacher duties, and coping with challenging interactions with students such as student misbehavior, lack of discipline in the classroom, and instances of violence and aggression. Additionally, interactions with parents represent an important job demand (Alcantara et al., 2019; De Ceballos and Carvalho, 2021; Hlado et al., 2020; Hlado and Harvankova, 2024; Sottimano et al., 2017; Vertanen-Greis et al., 2022).

In the qualitative study conducted among upper secondary school teachers (Hlado and Harvankova, 2024), prolonged exposure to job demands was associated not only with a decline in PTWA but also with the occurrence of burnout—a work-related psychological syndrome characterized by physical and emotional exhaustion, depersonalization, reduced personal accomplishment, and diminished professional efficacy (Maslach et al., 2001). Numerous studies, including those focused on teachers (Hakanen et al., 2006; Skaalvik and Skaalvik, 2020; Xanthopoulou et al., 2007), have established this link between job demands and burnout. However, the JD-R model and related research (Hlado et al., 2020) suggest that burnout may also operate as a mediator between job demands and perceived WA. The aforementioned study (Hlado and Harvankova, 2024), in line with the health impairment process (Taris et al., 2017), found that when teachers perceived job demands as excessively high or burdensome or when they encountered obstacles in meeting these demands, their job stress increased. Job-related distress, if not effectively managed, led among the teachers in this study to fatigue, exhaustion, and subsequently, physical and mental health issues, as well as a decline in PTWA (Hlado and Harvankova, 2024). In this light, we hypothesize:

- **Hypothesis 1**: Higher quantitative, cognitive, and emotional job demands are associated with (a) lower PTWA and (b) higher teacher burnout.
- **Hypothesis 2**: Burnout mediates the associations between quantitative, cognitive, and emotional job demands and PTWA.

Studies evaluating the limitations of the JD-R model highlight that this theory focuses exclusively on job-related aspects, neglecting the non-work domain (McGonagle et al., 2022). However, empirical evidence indicates that work and family are essential life domains, and achieving a balance between these two domains is crucial for teacher burnout and PTWA (Hlado et al., 2020; Hlado and Harvankova, 2024). The interconnection between work and family is supported by studies indicating that challenging job demands may lead to work-life conflicts on the one hand (Gu and Wang, 2021), while increased work-life conflicts are associated with higher levels of burnout and lower WA on the other (Abdelrehim et al., 2023; Bacci et al., 2024; Shields and Chen, 2021). However, generalizable findings regarding teachers are currently lacking. Drawing on the findings from a qualitative study among teachers (Hlado and Harvankova, 2024), work-life conflict may play a mediating role between job demands and burnout as well as perceived WA. Hlado and Harvankova's (2024) study revealed that teachers outside standard working hours frequently complete numerous tasks inherent to the teaching profession. As a result, this can contribute to work-life conflict, which may cause exhaustion and decrease their capacity to meet work requirements. Considering previous findings and the understanding that work and non-work domains are intricately intertwined and cannot be examined separately, we hypothesize that:

- **Hypothesis 3:** Higher levels of quantitative, cognitive, and emotional job demands are positively associated with increased teacher work-life conflict.
- **Hypothesis 4**: Work-life conflict is (a) positively associated with teacher burnout and (b) negatively associated with PTWA.
- **Hypothesis 5**: Work-life conflict mediates the relationships between quantitative, cognitive, and emotional job demands and (a) teacher burnout and (b) PTWA.

### The effect of job resources

In the present study, we examined three of the seven categories identified as job resources positively related to WA (Brady et al., 2020): supervisor support, coworker support, and job control, represented by autonomy. Social support at work, provided by supervisors and coworkers, encompasses various forms of assistance, including instrumental, informational, emotional, and appraisal support, that employees receive within organizational settings (Jolly et al., 2021). Such support can directly reduce job demands or make them seem less overwhelming, as evidenced by findings within the JD-R model (Demerouti et al., 2001; Schaufeli and Taris, 2014).

Research on diverse populations suggests that supportive organizational climates, positive supervisory and interpersonal relationships, constructive feedback, and both supervisor and coworker support can enhance WA (Ahlstrom et al., 2013; Airila et al., 2014; Bethge and Radoschewski, 2010; Boelhouwer et al., 2020; Feldt et al., 2009; Leijon et al., 2017; McGonagle et al., 2014). A study examining specific sources of social support among healthcare workers in Australia revealed that support provided by supervisors and coworkers significantly influences perceived WA (McGonagle et al., 2014). However, detailed insights into such effects among lower and upper secondary school teachers are lacking. The only available study, which focused on preschool teachers, revealed that coworker support alone has a significant positive impact on WA (Sottimano et al., 2017).

Research consistently highlights that supervisor and coworker support serve as vital resources that can alleviate the adverse effects of job demands. Specifically, supervisor and coworker support are associated with lower levels of burnout, particularly emotional exhaustion and depersonalization (Afota et al., 2024). Support from supervisors is recognized as a crucial resource for mitigating burnout across various professions, including the teaching profession (Leung and Lee, 2006).

In summary, support from supervisors and coworkers may play a crucial role in influencing teachers' levels of burnout and perceived WA, as well as their perceptions of job demands. Research findings, consistent with the JD-R model, suggest that social support at work serves as a job resource that may mitigate the negative effects of job demands while enhancing the capacity to meet these demands. Building on the theoretical and empirical arguments presented above, we propose the following hypotheses:

- **Hypothesis 6**: Higher levels of (a) supervisor support and (b) coworker support are associated with lower perceptions of quantitative, cognitive, and emotional job demands among teachers.
- **Hypothesis 7**: Higher levels of (a) supervisor support and (b) coworker support are associated with lower levels of teacher burnout.
- **Hypothesis 8**: Higher levels of (a) supervisor support and (b) coworker support are associated with higher levels of PTWA.
- **Hypothesis 9**: Quantitative, cognitive, and emotional job demands mediate the relationships between (a) supervisor support and PTWA and (b) coworker support and PTWA.

Autonomy, as a form of job control, refers to the extent to which individuals possess freedom, independence, and discretion in performing their work assignments (Morgeson and Humphrey, 2006). Although autonomy may present challenges related to responsibility for some teachers, thereby functioning as a job demand, research utilizing the JD-R model has previously recognized autonomy as a job resource (Taris et al., 2017). This construct includes the ability to schedule work, make decisions, and choose the methods and procedures used to perform tasks. Thus, autonomy can influence job demands by allowing teachers to tailor their job tasks and work plans to align with their strengths and abilities. When teachers have the autonomy to determine the content of their work, along with the methods and procedures they employ, it can either directly reduce job demands or lead them to perceive these demands as more manageable and less challenging. This perception, in turn, may help mitigate the adverse effect of job demands on burnout and PTWA.

Conceptually, autonomy empowers teachers to determine the most effective way to align their available job resources with current demands. Accordingly, a lack of autonomy has been found to be adversely related to burnout and WA (Boelhouwer et al., 2020; Guo et al., 2023; Tuomi, 2001; Van Den Berg et al., 2009). Furthermore, autonomy is considered a preventing factor concerning work-life conflict (Brauchli et al., 2014; Thompson and Prottas, 2006). Overall, autonomy may serve as a protective factor against work-life conflict, teacher burnout, and low PTWA, both directly and indirectly, through the management of job demands. Building on the theoretical and empirical arguments, we formulate the following hypotheses:

- **Hypothesis 10**: Autonomy in the teaching profession is negatively associated with (a) perceptions of quantitative, cognitive, and emotional job demands, (b) work-life conflict, and (c) burnout, while being positively associated with (d) PTWA.
- **Hypothesis 11**: Quantitative, cognitive, and emotional job demands mediate the relationship between autonomy and (a) work-life conflict, (b) burnout, and (c) PTWA.

**Figure 1:**
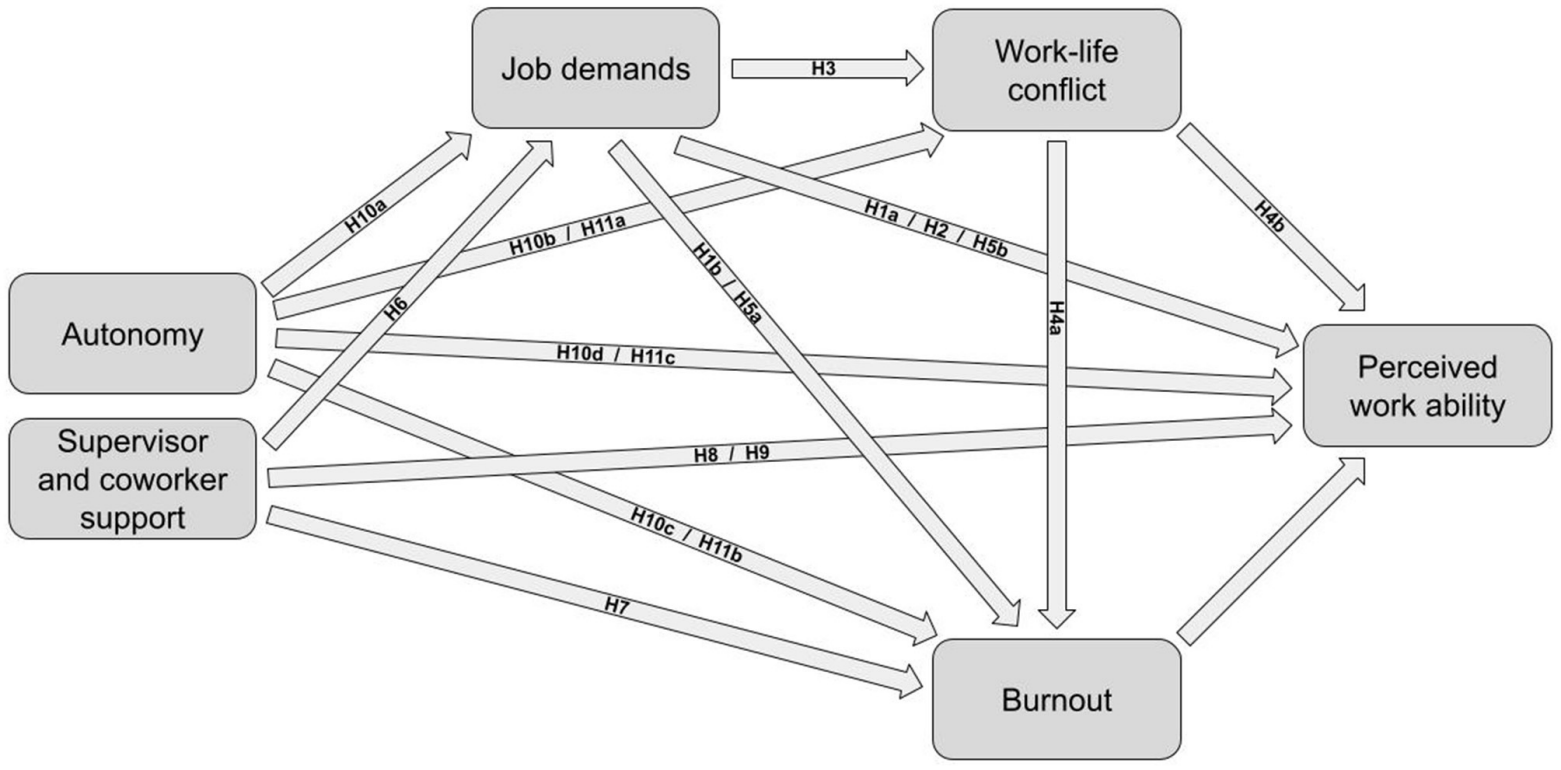
Proposed research model.

The proposed research model, which conceptualizes the relationships among job demands, job resources, work-life conflict, burnout, and PTWA, is presented in Figure 1.

## Methods

### Participants and procedure

A total of 352 schools providing primary and lower secondary education in two regions of the Czech Republic—South Moravian Region and Vysočina Region—were contacted via email. The school administrations of these schools were invited to participate in a longitudinal mixed-methods study. The consent was granted by 44 schools, forming the basis for this study.

This study draws on quantitative data collected during the first wave of data collection involving teachers from participating schools. Teachers were invited to participate in the research through a letter from the researchers, which was distributed by headteachers. However, the participation of the teaching staff in the research was voluntary. The data collection took place from October and November 2023 using an online questionnaire.

The sample comprises 841 primary and lower secondary teachers (86.1% female). Participants' ages ranged from 22 to 76 years, with a mean age of 45.9 years (*SD* = 10.8). On average, the teachers had 19.3 years of experience in the teaching profession (*SD* = 12.04), and 42.2% of them were teachers at the level of primary education (ISCED 1), while 54.1% were teachers at the level of lower secondary education (ISCED 2). For the remaining 3.7% of teachers, it was not possible to distinguish between primary and lower secondary education, or they worked at both levels. The participants taught various subjects, including biology, chemistry, languages, mathematics, physics, and physical education. The sample demonstrates the demographic representativeness of Czech teachers concerning age, years of teaching experience, and gender. Statistical data indicate that the average age of Czech teachers in primary and lower secondary education is 46 years, with an average teaching experience of 19 years (Czech School Inspectorate, 2023). Additionally, female teachers constitute a significant majority, representing 83% of the workforce (MEYS, 2024).

### Measures

**Perceived teacher work ability** was assessed using the Teacher Work Ability Scale (TWAS), a tool designed to measure teachers' perceived physical and mental capacity to meet various job demands within the teaching profession. The Czech version used in this study measured the perceived WA of teachers using 21 items. Teachers were asked, “Considering all the requirements of your teaching job, how do you rate your physical and mental capacity to meet these demands?” Responses were given on a 7-point Likert scale ranging from 1 (*low*) to 7 (*high*). The TWAS has a 5-factor structure consisting of the following dimensions: instructional management (5 items, an example item: “Keep students' attention in lesson”), teaching organization (4 items, an example item: “Organize team group work of students”), teacher-staff interaction (4 items, an example item: “Receive feedback from superiors and colleagues”), navigating difficult situations (3 items, an example item: “Address problematic situations with students”), and non-teaching responsibilities (5 items, an example item: “Supervise students outside the classroom”). The Cronbach's α for the TWAS in the sample was 0.95 for the whole scale, while the Cronbach's α for the individual dimensions were as follows: 0.88 for instructional management, 0.91 for teaching organization, 0.86 for teacher-staff interaction, 0.90 for navigating difficult situations, and 0.84 for non-teaching responsibilities. A second-order confirmatory factor analysis (CFA) was conducted to confirm the five-factor structure of the TWAS. The results of the CFA showed a good fit (χ^2^ |177| = 796.9, *p* < 0.001, CFI = 0.951, TLI = 0.942, RMSEA = 0.065, SRMR = 0.042), supporting the proposed five-factor structure of the TWAS.

**Burnout** was measured using the Czech version of the Shirom-Melamed Burnout Questionnaire (SMBQ; Ptáček et al., 2017). The SMBQ is a 14-item inventory consisting of three subscales that measure physical exhaustion (6 items), cognitive weariness (5 items), and emotional exhaustion (3 items), with high scores indicating high burnout. The SMBQ items were measured on a 7-point Likert-type scale with response options ranging from 1 (*never or almost never*) to 7 (*always or almost always*). In the present study, Cronbach's α of the total score was 0.93, and 0.92 for physical exhaustion, 0.88 for cognitive weariness, and 0.92 for emotional exhaustion subscales. A second-order CFA model with the presupposed three-factor structure showed a good fit (χ^2^ |73| = 274.5, *p* < 0.001, CFI = 0.977, TLI = 0.971, RMSEA = 0.057, SRMR = 0.036).

**Job resources, job demands, and work-life conflict** were assessed using the Czech version of the Copenhagen Psychosocial Questionnaire (COPSOQ-III; Burr et al., 2019), which is designed to assess various dimensions of the psychosocial factors at work. Job resources were measured by three different scales: social support from supervisor (3 items, an example item: “How often is your immediate superior willing to listen to your problems at work, if needed?,” Cronbach's α = 0.82), social support from colleagues (3 items, an example item: “How often are your colleagues willing to listen to your problems at work, if needed?,” Cronbach's α = 0.77), and influence at work measuring autonomy at work (5 items, an example item: “Can you influence the amount of work assigned to you?,” Cronbach's α = 0.75). Within job demands, three different dimensions of demands were distinguished and measured: quantitative job demands (4 items, an example item: “Do you get behind with your work?,” Cronbach's α = 0.77), cognitive job demands (4 items, an example item: “Does your work require you to make difficult decisions?,” Cronbach's α = 0.75), emotional job demands (4 items, an example item: “Does your work put you in emotionally disturbing situations?,” Cronbach's α = 0.76). The work-life conflict was measured using the work-life conflict scale (3 items, an example item: “Do you feel that your work drains so much of your energy that it has a negative effect on your private life?,” Cronbach's α = 0.82). The long version of the COPSOQ-III scales was used to measure all variables. The questionnaire items were rated on Likert-type scales with five response options, ranging from 1 (*never*) to 5 (*always*). The CFA performed on all seven scales showed a good fit (χ^2^ |250| = 832.5, *p* < 0.001, CFI = 0.926, TLI = 0.911, RMSEA = 0.053, SRMR = 0.051), thus supporting the ability of the chosen instrument to discriminate and measure the individual dimensions of job resources, job demands, and work-related conflict.

### Data analysis

Prior to the main analysis, a confirmatory factor analysis (CFA) was used to assess the psychometric properties of all measures used in this study and to confirm their expected factor structure. In addition, Cronbach's alpha was calculated for all measures to assess their reliability. In order to estimate the proposed model and test the above hypotheses, structural equation modeling (SEM) was used, applying the maximum likelihood estimation method. The analyses were carried out in the R statistical environment (R Core Team, 2023), relying particularly on the *lavaan* package (Rosseel, 2012). To test the full SEM model as well as to assess the goodness of fit of the CFA models, several measures of fit were used and reported in the study. Specifically, we used: χ^2^ (chi-squared), comparative fit index (CFI), Tucker-Lewis index (TLI), root mean square error of approximation (RMSEA), and standardized root mean square residual (SRMR). However, as the χ^2^ indicator is sensitive to sample size (Schumacker and Lomax, 2010), this indicator was not interpreted strictly, and we focused on the remaining indicators to evaluate the goodness of fit of the CFA models. The following recommendations were followed to assess the goodness of fit of the models: The measures of CFI and TLI indicated an acceptable fit with a threshold above 0.90 and a good fit above 0.95. The measures of RMSEA and SRMR value indicated an acceptable fit below 0.08 and a good fit below 0.05 (Hu and Bentler, 1999; Kline, 2016).

## Results

We developed and estimated the full SEM model based on the proposed hypotheses. The structural model results yielded the following goodness-of-fit indices: χ^2^ |1,656| = 4,210.5, *p* < 0.001, CFI = 0.918, TLI = 0.912, RMSEA = 0.043, SRMR = 0.050. Comparing these values to the critical thresholds outlined earlier, the CFI and TLI exceed the recommended cutoff of 0.90. Similarly, both the RMSEA and SRMR are below the acceptable threshold of 0.08, with the RMSEA even falling below the more stringent criterion of 0.05. These findings indicate that the proposed conceptual model demonstrates an acceptable fit to the empirical data. Regarding hypothesis testing, the main results are summarized in Table 1 (direct effect hypotheses) and Table 2 (indirect effect hypotheses). A detailed explanation of these findings is provided below.

**Table 1:** Direct effects for the proposed SEM model.

	**Est**	**CI**	***p***
Autonomy → Quantitative job demands	−0.131	[−0.235; −0.028]	**0.013**
Supervisor support → Quantitative job demands	−0.123	[−0.227; −0.019]	**0.020**
Coworker support → Quantitative job demands	−0.025	[−0.125; 0.074]	0.621
Autonomy → Cognitive job demands	0.154	[0.052; 0.257]	**0.003**
Supervisor support → Cognitive job demands	−0.124	[−0.228; −0.019]	**0.021**
Coworker support → Cognitive job demands	0.096	[−0.004; 0.196]	0.059
Autonomy → Emotional job demands	−0.110	[−0.211; −0.010]	**0.031**
Supervisor support → Emotional job demands	−0.083	[−0.185; 0.020]	0.114
Coworker support → Emotional job demands	0.017	[−0.081; 0.114]	0.738
Autonomy → Work–life conflict	−0.152	[−0.226; −0.079]	**<0.001**
Quantitative job demands → Work–life conflict	0.493	[0.422; 0.564]	**<0.001**
Cognitive job demands → Work–life conflict	0.047	[−0.049; 0.143]	0.334
Emotional job demands → Work–life conflict	0.252	[0.156; 0.349]	**<0.001**
Supervisor support → Burnout	−0.082	[−0.166; 0.001]	0.054
Coworker support → Burnout	−0.099	[−0.178; −0.019]	**0.015**
Autonomy → Burnout	−0.035	[−0.124; 0.055]	0.445
Quantitative job demands → Burnout	0.388	[0.288; 0.488]	**<0.001**
Cognitive job demands → Burnout	−0.138	[−0.239; −0.037]	**0.007**
Emotional job demands → Burnout	0.202	[0.097; 0.307]	**<0.001**
Work–life conflict → Burnout	0.285	[0.183; 0.387]	**<0.001**
Work–life conflict → PTWA	0.037	[−0.073; 0.147]	0.508
Autonomy → PTWA	0.053	[−0.039; 0.146]	0.258
Quantitative job demands → PTWA	−0.179	[−0.302; −0.057]	**0.004**
Cognitive job demands → PTWA	0.105	[−0.002; 0.212]	0.054
Emotional job demands → PTWA	0.022	[−0.092; 0.136]	0.706
Supervisor support → PTWA	0.091	[0.004; 0.178]	**0.041**
Coworker support → PTWA	0.152	[0.069; 0.235]	**<0.001**
Burnout → PTWA	−0.368	[−0.492; −0.244]	**<0.001**

Significant effects are shown in bold.

**Table 2:** Indirect effects for the proposed SEM model.

	**Est**	**CI**	***p***
**Work-life conflict as a dependent variable**			
Autonomy → Quantitative job demands	−0.065	[−0.116; −0.013]	**0.013**
Autonomy → Cognitive job demands	0.007	[−0.008; 0.023]	0.366
Autonomy → Emotional job demands	−0.028	[−0.055; −0.001]	**0.043**
**Burnout as a dependent variable**			
Quantitative job demands → Work–life conflict	0.140	[0.089; 0.192]	**<0.001**
Cognitive job demands → Work–life conflict	0.013	[−0.015; 0.042]	0.350
Emotional job demands → Work–life conflict	0.072	[0.035; 0.109]	**<0.001**
Autonomy → Quantitative job demands	−0.051	[−0.093; −0.009]	**0.018**
Autonomy → Cognitive job demands	−0.021	[−0.042; −0.001]	**0.048**
Autonomy → Emotional job demands	−0.022	[−0.046; 0.001]	0.061
**PTWA as a dependent variable**			
Quantitative job demands → Burnout	−0.143	[−0.202; −0.083]	**<0.001**
Cognitive job demands → Burnout	0.051	[0.010; 0.091]	**0.014**
Emotional job demands → Burnout	−0.074	[−0.121; −0.028]	**0.002**
Quantitative job demands → Work–life conflict	0.018	[−0.036; 0.073]	0.510
Cognitive job demands → Work–life conflict	0.002	[−0.004; 0.008]	0.579
Emotional job demands → Work–life conflict	0.009	[−0.019; 0.037]	0.511
Supervisor support → Quantitative job demands	0.022	[−0.002; 0.046]	0.073
Supervisor support → Cognitive job demands	−0.013	[−0.030; 0.004]	0.139
Supervisor support → Emotional job demands	−0.002	[−0.011; 0.008]	0.713
Coworker support → Quantitative job demands	0.004	[−0.014; 0.023]	0.625
Coworker support → Cognitive job demands	0.010	[−0.006; 0.025]	0.174
Coworker support → Emotional job demands	< 0.001	[−0.002; 0.003]	0.800
Autonomy → Quantitative job demands	0.024	[−0.001; 0.048]	0.060
Autonomy → Cognitive job demands	0.016	[−0.003; 0.036]	0.107
Autonomy → Emotional job demands	−0.002	[−0.015; 0.010]	0.710

Significant effects are shown in bold.

Hypothesis 1 proposed that higher job demands would be associated with lower PTWA and, at the same time, with higher teacher burnout. The results of the SEM model only partially supported *Hypothesis 1*. Specifically, quantitative job demands were significantly associated with lower PTWA, while cognitive and emotional job demands showed no significant associations. Regarding the relationship with teacher burnout, all three job demands were significantly associated with burnout. However, contrary to expectations, higher cognitive job demands were linked to lower levels of teacher burnout rather than higher levels, as initially hypothesized. With respect to Hypothesis 2, we proposed that burnout mediates the relationship between job demands and PTWA. As shown in Table 2, *Hypothesis 2* was fully supported. Specifically, a statistically significant indirect effect of burnout on PTWA was observed for all three types of job demands. These findings confirm that burnout plays a significant intermediary role in the relationship between job demands and PTWA. However, it is important to note that, in the case of cognitive job demands, the direction of the relationship differs from that of quantitative and emotional job demands. This result is also contrary to what would typically be expected based on existing research and theoretical frameworks in this area.

Hypothesis 3 posited that higher levels of job demands would be associated with an increased risk of work-life conflict among teachers. *Hypothesis 3* was only partially supported by the results. Specifically, quantitative and emotional job demands were significantly and positively associated with work-life conflict, whereas cognitive job demands showed no significant relationship with work-life conflict. Hypothesis 4 examined the relationships between work-life conflict and teacher burnout, as well as between work-life conflict and PTWA. Specifically, the hypothesis posited a positive association in the case of teacher burnout and a negative association in the case of PTWA. The results of the estimated SEM model indicate that only the first part of *Hypothesis 4* is supported by our data. Specifically, work-life conflict is significantly and positively associated with teacher burnout but shows no significant relationship with PTWA. Following on from the direct effects, Hypothesis 5 examined whether work-life conflict mediates the relationship between job demands and, respectively, teacher burnout and PTWA. The results provided partial support for the first part of *Hypothesis 5*, i.e., the indirect effect of job demands on burnout through work-life conflict. Work-life conflict significantly mediates the relationship between quantitative and emotional job demands and burnout. However, it does not mediate the relationship between cognitive job demands and burnout. Regarding the relationship between job demands and PTWA, no significant indirect effect of work-life conflict was observed.

The remaining hypotheses examined the role of work resources in the relationships under investigation. Hypotheses 6 to 9 focused on support from supervisors and coworkers, while Hypotheses 10 and 11 addressed autonomy as a job resource. Hypothesis 6 examined whether higher levels of perceived supervisor and coworker support are associated with lower levels of perceived job demands. The results partially supported *Hypothesis 6*, as a statistically significant association was found only for supervisor support, with no such association observed for coworker support. This indicates that perceptions of coworker support were not related to perceptions of job demands. In addition, supervisor support was only significantly related to quantitative and cognitive job demands but not to emotional ones. Hypothesis 7 proposed that higher levels of supervisor and coworker support would be associated with lower levels of teacher burnout, while Hypothesis 8 posited that higher levels of supervisor and coworker support would be associated with higher levels of PTWA. *Hypothesis 7* was only partially supported, with support from coworkers showing a significant relationship. Although the *p*-value for supervisor support was relatively close to the 0.05 threshold, it suggests that supervisor support may still be an important factor in relation to teacher burnout. *Hypothesis 8* was fully supported, highlighting the positive impact of both types of support on teachers' perceptions of their WA. *Hypothesis 9*, which examined the potential mediating effect of job demands on the relationships between supervisor and coworker support and PTWA, was not supported. This indicates no significant indirect effects of supervisor and coworker support on PTWA through job demands.

Hypothesis 10 focused on teacher autonomy and examined its associations with four other variables. *Hypothesis 10a* proposed a negative association between autonomy and job demands and was partially supported. Specifically, autonomy was negatively associated with both quantitative and emotional job demands. However, contrary to the hypothesis, a positive association was found with cognitive job demands. Hypothesis 10b proposed a negative relationship between autonomy and work-life conflict. The results supported *Hypothesis 10b*, indicating that higher levels of teacher autonomy were associated with a lower risk of work-life conflict. Hypothesis 10c proposed a negative relationship between autonomy and burnout, while Hypothesis 10d posited a positive relationship between autonomy and PTWA. Neither *Hypothesis 10c* nor *Hypothesis 10d* was supported, indicating no significant direct link between autonomy and burnout or between autonomy and PTWA.

Finally, Hypothesis 11 examined whether the relationships between teacher autonomy and the other variables (i.e., work-life conflict, burnout, and PTWA) were mediated by job demands. This hypothesis was not supported in relation to the relationship between autonomy and PTWA, suggesting no significant mediating effects of job demands in this pathway, and therefore, *Hypothesis 11c* was not supported. However, partial support was found for the relationships between autonomy and work-life conflict (*Hypothesis 11a*) and between autonomy and burnout (*Hypothesis 11b*). In the first case, quantitative and emotional job demands significantly mediate the relationship between autonomy and work-life conflict. In the second case, quantitative and cognitive job demands significantly mediate the relationship between autonomy and burnout, with the *p*-value for emotional job demands being relatively close to the 0.05 threshold.

## Discussion

This was the first study to comprehensively examine the antecedents of PTWA, including the relationships between teacher job demands (quantitative, qualitative, and emotional), job resources (supervisor support, coworker support, and autonomy), and burnout, using structural equation modeling. In contrast to the traditional JD-R model (Schaufeli and Taris, 2014), our research model was expanded to include the non-work domain to investigate PTWA. This expansion was informed by findings, which demonstrated that the work and non-work domains are intricately intertwined and cannot be examined separately, particularly when investigating perceived WA (Hlado and Harvankova, 2024; McGonagle et al., 2022). Our findings provided mixed support for WA literature (Brady et al., 2020; Cadiz et al., 2019; Hlado and Harvankova, 2024) and the JD-R model (Bakker and Demerouti, 2007; Demerouti and Bakker, 2011; Schaufeli and Taris, 2014; Taris et al., 2017) among a sample of Czech teachers, and they are further discussed with a particular emphasis on PTWA.

While our study did not explicitly formulate a hypothesis regarding the relationship between burnout and PTWA, burnout emerged as the most statistically significant antecedent of PTWA, demonstrating a strong negative effect. This outcome is consistent with prior research that has similarly recognized burnout as a key predictor among teachers (Hlado et al., 2020). Consequently, alongside PTWA, we will place increased emphasis on the findings related to teacher burnout.

The teaching profession is known for having many job demands. In line with expectations (Hakanen et al., 2006; Skaalvik and Skaalvik, 2020; Xanthopoulou et al., 2007), all three types of job demands had a direct effect on teacher burnout. However, an unexpected finding was that higher cognitive demands were linked to lower burnout levels. This result diverges from the general assumptions of the JD-R model (Taris et al., 2017), which posits that excessive job demands typically lead to adverse outcomes and deplete individuals' energy and psychological resources, thereby increasing the risk of burnout. However, cognitive demands likely function as a challenge rather than a hindrance stressors for teachers. Challenge stressors, such as solving problems, engaging in intellectually stimulating tasks, and adapting to diverse needs typical of the teaching profession, can serve as motivational factors and provide opportunities for personal growth, learning, and mastery (Podsakoff et al., 2007). Empirical studies have shown that when individuals perceive cognitive demands as meaningful and manageable, these demands can promote engagement and even reduce the risk of burnout by enhancing intrinsic motivation and fostering a sense of competence (Rodell and Judge, 2009).

Furthermore, only quantitative job demands were directly and negatively associated with PTWA. Regarding cognitive and emotional demands in the teaching profession, these were not linked to PTWA. Our results may be attributed to a professional effect; teachers who choose this career may expect to encounter cognitive and emotional demands as an inherent aspect of their work, which might prevent them from viewing these job demands as burdensome or exhausting (McGonagle et al., 2014). Based on research (Schaufeli and Taris, 2014), many teachers develop adaptive strategies to manage these job demands, potentially reducing their impact on PTWA. Another possible explanation for the lack of a direct link between cognitive and emotional job demands in the teaching profession and reduced PTWA may lie in their dual nature, as described earlier. Our findings suggest that teachers might perceive cognitive and emotional job demands as challenges that provide opportunities for personal growth, learning, and development (Hlado and Harvankova, 2024; McGonagle et al., 2014), which could reduce the risk of a decline in PTWA. Additionally, teachers who lack the resources to manage cognitive and emotional job demands may leave the profession and thus were not represented in our study. However, these potential explanations highlight the need for further exploration.

Consistent with previous research (Hlado et al., 2020; Hlado and Harvankova, 2024), our results suggest that job demands indirectly affect PTWA. All three types of job demands—quantitative, cognitive, and emotional—exerted a mediating effect on PTWA through burnout. The present study extends the understanding of the health impairment process proposed in the JD-R model (Taris et al., 2017), emphasizing that high quantitative and emotional job demands in the teaching profession are associated with physical and psychological costs. These costs can deplete energy and lead to burnout, adversely affecting not only teachers' health, as shown in several studies (Hakanen et al., 2006) but also other work-related outcomes, such as reduced PTWA, as demonstrated in the present study. Our findings are important not only because cognitive job demands may play a protective role against burnout but also because they may indirectly enhance PTWA through their impact on burnout. From the perspective of the JD-R model (Taris et al., 2017), it seems that cognitive job demands may transform into job resources in the teaching profession, as they likely have inherent motivational qualities. These findings also require further research to uncover the underlying mechanism behind it.

The inclusive framework we proposed, encompassing work and non-work domains, revealed that high quantitative and emotional job demands may contribute to work-life conflict. Contrary to expectations (Gu and Wang, 2021; Hlado and Harvankova, 2024), the results show that cognitive job demands do not necessarily lead to work-life imbalance and related work-life conflict. This may suggest that teachers have adequate resources for managing cognitive job demands, unlike quantitative and emotional job demands, which disrupt their family life (Gu and Wang, 2021). One potential explanation is that, with increasing work experience, teachers may develop more effective strategies for managing cognitive job demands or perceive them as less intensive, thereby mitigating their impact on work-life conflict (Kubicek et al., 2022). Furthermore, our results support the previous findings that work-life conflict contributes to teacher burnout (Shields and Chen, 2021). In contrast to the previous findings (Abdelrehim et al., 2023; Bacci et al., 2024; Berglund et al., 2021), we did not find that work-life conflict directly affects PTWA. Despite this, work-life conflict remains an important variable in our model, as it influences burnout, a key antecedent of PTWA. In the present study, we did not examine the mediating effect of burnout in the relationship between work-life conflict and PTWA, which was suggested by a previous qualitative study on teachers (Hlado and Harvankova, 2024). We recommend that further research focus on this aspect.

Our expectation that work-life conflict mediates the relationship between quantitative, cognitive, and emotional job demands, and both burnout and PTWA was only partially supported. Specifically, work-life conflict acted as a mediator solely in the relationship between quantitative and emotional demands and burnout. Nevertheless, these findings extend existing literature which has mainly focused on the direct relationships between job demands and work-life conflict (Gu and Wang, 2021), as well as between work-life conflict and burnout (Shields and Chen, 2021) and between work-life conflict and WA (Abdelrehim et al., 2023; Bacci et al., 2024). As previous research on teachers has shown (Hlado and Harvankova, 2024), to manage quantitative job demands, teachers often extend their work beyond regular hours. Our study indicates that a similar mechanism could apply to the emotional demands teachers need to manage, which can also impact them beyond working hours. Overall, high quantitative and emotional job demands in the teaching profession can negatively affect family relationships, the fulfillment of non-work commitments, and active and passive rest. Our finding that quantitative and emotional job demands indirectly contribute to teacher burnout through work-life conflict suggests that if teachers cannot manage these demands effectively, it negatively demonstrates in work-life conflict which causes fatigue and consequently burnout. The present study, therefore, validates the qualitative findings obtained from in-depth interviews conducted with teachers (Hlado and Harvankova, 2024). In summary, the present findings indicate that work and life are essential for teachers, and maintaining a balance between these two is crucial for preventing burnout. However, this balance shows no significant effect on PTWA.

In line with the JD-R model (Demerouti et al., 2001; Schaufeli and Taris, 2014) and previous research across different professional groups (Afota et al., 2024; Alavinia et al., 2009; Brauchli et al., 2014; Guo et al., 2023; Leung and Lee, 2006; McGonagle et al., 2014; Sottimano et al., 2017), our study showed that job resources such as supervisor and coworker support, as well as autonomy, can not only alleviate teachers' perceptions of job demands and reduce the risk of burnout but also mitigate work-life conflict and improve PTWA. The present study contributes significantly to the literature by demonstrating how the influence of job resources on job demands, burnout, and PTWA differs depending on the resource type, thereby providing empirically grounded insights for potential interventions.

As anticipated, we found that supervisor support has a direct negative effect on quantitative and cognitive job demands among teachers and contributes to improving PTWA. The results of our study, which show that supervisor support leads to improved PTWA, align with previous research (Boelhouwer et al., 2020; McGonagle et al., 2014). Contrary to our expectations, the influence of supervisor support on emotional job demands and teacher burnout was not confirmed. This result differs from the assumptions of the JD-R model (Bakker et al., 2005) and prior research (Afota et al., 2024; Leung and Lee, 2006) that has demonstrated a negative association between supervisor support and burnout. Our findings related to the role of supervisor support in the teaching profession underscore the importance of enhancing supervisor support as a key resource, particularly for fostering PTWA and shaping the perception of quantitative and cognitive job demands. A critical issue that emerged in our research is how headteachers, as school leaders, could contribute to preventing burnout and supporting the emotional job demands of teachers, which were in our research linked to work-life conflict and burnout.

According to the literature review, coworker support serves as another important job resource in the JD-R model (Demerouti et al., 2001; Schaufeli and Taris, 2014). Based on our results, coworker support helps mitigate the risk of burnout and enhances PTWA but does not have a direct effect on the perception of job demands. On the other hand, insufficient or lack of coworker support may thus negatively manifest in energy depletion and the development of burnout, as well as in reduced capacity to meet job demands. Our findings regarding the substantial influence of coworker support on burnout and PTWA are, to some extent, explainable by the specific nature of relationships within the teaching profession. In Czech schools, teachers share their workspaces daily with colleagues in staff rooms or common areas, which creates opportunities for building intense, deep, and often close relationships. Teachers have the chance to share their challenges at work and offer advice and support, which can manifest in lower burnout risk and higher PTWA. In light of this explanation, it is therefore surprising that instrumental and psychosocial support from colleagues does not manifest in the perception of quantitative, qualitative, and emotional job demands in our study.

Although supervisor support was associated with the perception of specific job demands, the findings related to coworker support raise concerns about the interaction effect between job demands and job resources, as introduced in the revised JD-R model in 2007 (Bakker and Demerouti, 2007). Nevertheless, our finding aligns with prior studies, where less than two-thirds of the 50 tested interactions between job demands and resources were significant (Taris et al., 2017). Further research appears essential to uncover the mechanisms underlying why some job resources interact with job demands among teachers, whereas others fail to, enabling the effective use of job resources to address the challenges of the teaching profession.

The third key job resource, autonomy, included in our investigation (Brady et al., 2020), has a direct negative effect on the perception of quantitative and emotional job demands. Therefore, promoting autonomy emerges as a potential strategy to support teachers in managing these job demands. On the other hand, our findings show that higher autonomy is linked to increased cognitive demands in the teaching profession. A potential reason is that cognitive demands promote professional growth (Clarà et al., 2022). Teachers with increased autonomy may place higher cognitive job demands on themselves to foster their professional development.

Additionally, we found that higher autonomy helps mitigate work-life conflict, which aligns with previous findings across various professions (Brauchli et al., 2014; Thompson and Prottas, 2006). Contrary to expectations (Boelhouwer et al., 2020; Guo et al., 2023; Tuomi, 2001; Van Den Berg et al., 2009), however, autonomy does not have a significant direct effect on teacher burnout or PTWA. Autonomy influences work-life conflict also indirectly through quantitative and emotional job demands. In line with expectations based on the literature and previous research, a mediating role of quantitative and cognitive job demands in the relationship between autonomy and burnout was also demonstrated. This suggests that autonomy in the teaching profession can serve as a protective factor for work-life conflict and teacher burnout, as it allows for individual adjustment and management of these job demands.

In our study, we hypothesized that supervisor support, coworker support, and autonomy would reduce job demands and thereby indirectly support PTWA. However, our assumptions were not confirmed. Nevertheless, our findings clearly demonstrated that a supportive organizational climate, in the form of supervisor and coworker support, is significant and indispensable for PTWA.

### Limitations and practical implications

First, this study employed a cross-sectional research design, which restricts the ability to draw causal inferences and assess long-term relationships. Second, our findings are based on self-reported scales, reflecting teachers' perceptions and evaluations of themselves, job demands, job resources, work-life conflict, burnout, and PTWA. As a result, the responses could be influenced by biases such as social desirability or inaccurate self-assessments, particularly when assessing variables like burnout, work-life conflict, and health status, which is an integral component of PTWA (Latkin et al., 2017). Moreover, self-reported data may not fully capture objective realities, as perceptions can differ significantly from observable behaviors or conditions. Third, the study was limited to a relatively homogeneous sample of Czech primary and lower secondary school teachers. This limits the generalizability of the findings to other groups, such as preschool and upper secondary school teachers, teaching assistants, and headteachers, as well as to different cultural contexts or professional populations. Our sample is also specific due to the significant proportion of female participants (86.1%), which aligns with the proportion of female teachers in the Czech education system (MEYS, 2024). However, women are also over-represented among primary and secondary education teachers in other OECD countries (OECD, 2021). This broader characteristic of educational systems implies that, with certain limitations, the findings may apply to the entire teacher population, including both men and women. As noted, perceived WA in teachers is associated with multiple aspects. Fourth, we focused only on selected job demands and job resources, work-life conflict, and burnout, which were highlighted in recent studies and found to be significant predictors of WA in teachers. It is also apparent that other risk factors may be associated with WA, such as physical and mental health. Another limitation is that our model did not include personal resources (e.g., self-esteem, self-efficacy, resilience, hope, and value orientation). Personal resources, which were more recently added to the JD-R model (Bakker and Demerouti, 2017), may also play a vital role in this dynamic. They can buffer the adverse effects of job demands and enhance the positive impact of job resources, thereby promoting higher levels of PTWA.

Despite these limitations, the present study has important practical implications. Several strategies can be employed to support PTWA. PTWA can be enhanced through strengthening social support, which involves, for example, creating strong networks among teaching staff where teachers can share resources, advice, and best practices. An effective approach is, for example, teacher sharing. Headteachers themselves, as key resources, can play a significant role by providing constructive feedback, creating mentoring programs, and facilitating communication among staff members. Another potential approach to supporting work ability, arising from our findings, is adjusting quantitative job demands. This includes, for example, personalizing workloads through flexible scheduling and considering individual circumstances when setting performance targets. Headteachers can also assist by reducing administrative burdens and providing additional support to teachers facing high demands. Furthermore, proactive support for PTWA can involve offering professional development opportunities and ensuring teachers have access to mental health resources. The prevention of teacher burnout should also be considered. Our research highlights the complex interplay of antecedents and a range of hidden relationships between them. While we have suggested potential pathways for supporting PTWA, it is also essential to promote teacher autonomy and implement measures within schools to prevent the emergence of work-life conflict.

## Conclusions

The present study examined the antecedents of PTWA within the framework of both work and non-work domains. The analysis identified quantitative job demands, supervisor and coworker support as job resources, and burnout as the most influential factors affecting PTWA. Among these, burnout was found to be the strongest negative predictor of PTWA. Furthermore, all examined job demands, along with work-life conflict, were significant predictors of burnout. While autonomy does not directly influence PTWA, it indirectly mitigates quantitative, cognitive, and emotional job demands, which in turn reduce burnout and enhance PTWA. Our findings emphasize the importance of a supportive and autonomous organizational climate, the management of job demands in accordance with individual capacities, and the implementation of targeted burnout prevention strategies to promote PTWA and maintain teachers' long-term professional engagement. Further research is needed to explore the dynamic interaction between work and non-work factors and to assess potential intervention strategies more comprehensively.

## Data Availability

The raw data supporting the conclusions of this article will be made available by the authors, without undue reservation.
